# Immune cell–adipose tissue crosstalk in metabolic diseases with a focus on type 1 diabetes

**DOI:** 10.1007/s00125-025-06437-z

**Published:** 2025-06-04

**Authors:** Fawaz Alzaid, Guy Fagherazzi, Jean-Pierre Riveline, Fatemah Bahman, Fatema Al-Rashed, Fahd Al-Mulla, Rasheed Ahmad

**Affiliations:** 1 Dasman Diabetes Institute, Kuwait City, Kuwait; 2 INSERM UMR-S1151, CNRS UMR-S8253, Université Paris Cité, Institut Necker Enfants Malades, Paris, France; 3 Deep Digital Phenotyping Research Unit, Department of Precision Health, Luxembourg Institute of Health, Strassen, Luxembourg; 4 Université Paris Cité, Department of Diabetology, Endocrinology and Nutrition, Lariboisière Hospital, GHU AP-HP. Nord, Paris, France

**Keywords:** Adipose tissue, Epidemiology, Immune cells, Obesity, Review, Translational, Type 1 diabetes

## Abstract

Adipose tissue, once regarded merely as an energy reservoir, has emerged as a critical regulator of both metabolic and immune processes. This paradigm shift has profound implications for understanding and managing type 1 diabetes, a condition typically associated with lean individuals. The growing global prevalence of obesity has introduced an underexplored dimension to type 1 diabetes pathophysiology, a phenomenon that has significant consequences for disease development, progression and management. The coexistence of obesity and type 1 diabetes presents unique challenges, including exacerbation of insulin resistance and an elevated risk of complications such as CVD. Obesity-induced chronic low-grade inflammation, or ‘meta-inflammation’, creates a proinflammatory environment within adipose tissue. This disrupts systemic immune regulation, promotes insulin resistance and may even potentiate autoimmunity directed to pancreatic beta cells. Addressing these interactions will allow us to reframe research priorities and the management of type 1 diabetes in individuals who also live with obesity. In this review, we explore how adipose tissue maladaptation in obesity influences the pathophysiology of type 1 diabetes. We discuss existing literature and gaps in knowledge, and emphasise the importance of addressing these gaps. We also highlight the potential of emerging technologies and precision medicine to tackle the dual challenge of obesity and type 1 diabetes. Advances such as continuous glucose monitoring and automated insulin delivery systems and insights from genomics and metabolomics are revolutionising diabetes care. These tools can enhance glucose management and provide opportunities to mitigate weight-related complications and personalise treatment strategies.

**Graphical Abstract:**

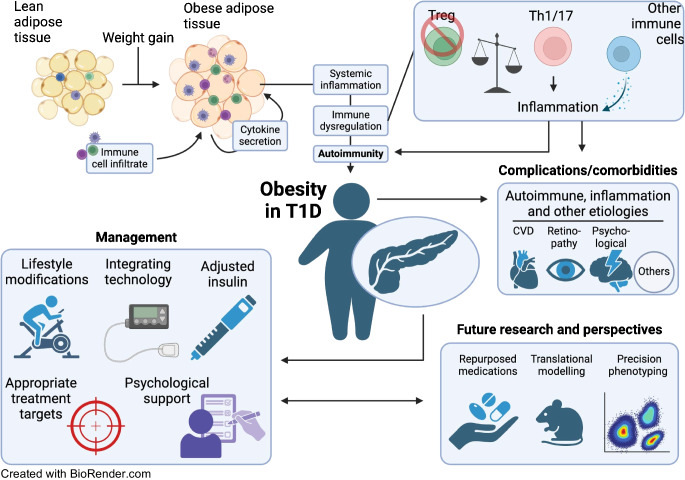

**Supplementary Information:**

The online version contains peer-reviewed but unedited supplementary material including a slideset of the figures for download, available at 10.1007/s00125-025-06437-z.

## Introduction

Adipose tissue, historically considered a relatively inert long-term energy store, is now recognised to dynamically contribute to several immune and metabolic disorders. Scientific and medical communities also recognise functional and phenotypic diversity in adipose tissue based on depot location and type, as well as across developmental and disease states. Many depots exist; the most widely studied are subcutaneous, visceral and supraclavicular, with the last being a depot of brown adipose tissue (BAT), only confirmed to exist in adult humans in 2009 [1]. Functionally, subcutaneous white adipose tissue (WAT) serves as an insulator, visceral WAT serves as a cushion around organs and as an energy store, and BAT generates heat (thermogenesis) [2].

Visceral WAT has gained attention for its roles in diseases with inflammatory or immune aetiologies, including diabetes. In type 2 diabetes, WAT has an established, well-defined role in chronic low-grade inflammation and insulin resistance [3, 4]. However, how adipose tissue maladaptation contributes to type 1 diabetes pathogenesis or pathophysiology is an emerging field.

Studies since the 1970s have hinted at the immune reactivity of WAT, and more recent research has redefined WAT as an energetically adaptable immune organ [5–7]. Both adipocytes and cells of the stromal vascular fraction (SVF), containing leukocytes, express immunologically active molecules that impact whole-body inflammatory status and metabolism. The most widely known cytokines, such as IL-6 and TNF, are mainly expressed by adipose tissue macrophages (ATMs). Their expression has also been reported in adipocytes at lower levels and in other SVF cells [8]. Although macrophages are recognised as the main source, studies investigating deletion of cytokine-coding genes in myeloid cells support an important role for non-myeloid sources [9]. For TNF, myeloid-specific silencing did not alter obesity-associated metabolic dysfunction despite lower circulating and tissue levels of the cytokine [9]. Other studies have demonstrated that the effects of a given cytokine on adipose tissue biology depend on the cell type expressing it. Notably, adipocyte-derived IL-6 increases macrophage infiltration, while myeloid- or muscle-derived IL-6 decreases it [10]. The role of IL-6 itself has been debated, as it has been shown to play both protective and deleterious roles. Nonetheless, adipose tissue is immunocompetent and has the capacity to produce inflammatory cytokines [11].

Many studies have characterised the adipose tissue immune niche and the reactivity of adipocytes to immune signalling [5, 12]. These studies primarily address innate immunity and have been in the areas of insulin resistance and type 2 diabetes. Of relevance to type 1 diabetes are the fewer, more recent, yet influential studies on adipose tissue adaptation to obesity and both local and systemic events that contribute to autoimmune mechanisms and the autoimmune form of diabetes [13, 14]. Interest in adipose tissue biology in type 1 diabetes is driven by epidemiological and genetic studies that highlight the increased risk of autoimmune conditions, including type 1 diabetes, in individuals with overweight or obesity [15, 16]. The relevance of this topic is also shown by epidemiological evidence of the increasing prevalence of obesity and type 1 diabetes [13, 17, 18].

In this review, we address immune cell–adipose tissue crosstalk in type 1 diabetes. Recent advances in translational research, technology, management and therapy are discussed. We highlight the links between adipose tissue (dys)function and type 1 diabetes to provoke a re-evaluation of research priorities and management modes, and targets, in type 1 diabetes. This includes consideration of how obesity influences the disease course and presents unique management challenges.

## Obesity and type 1 diabetes: definitions and epidemiology

Since 1980, the global prevalence of obesity has more than doubled, with obesity contributing to around 5 million deaths in 2019 (see electronic supplementary material [ESM], Text box: Key facts on obesity). Diabetes is a frequent contributing factor to these deaths [19, 20]. Diabetes has seen a fourfold increase in prevalence since 1980, with 537 million people living with diabetes in 2021 [21]. Type 1 diabetes, traditionally associated with a younger age of onset and a leaner body type, makes up 5–10% of all diabetes cases [21]. Epidemiological data from 2023–2024 indicate that the European region has the highest burden of type 1 diabetes worldwide and, in the USA, about 5.6% of adult diabetes cases are type 1 diabetes [22–24].

Emerging evidence suggests that additional factors, including obesity and lifestyle factors, impact the incidence and course of type 1 diabetes beyond autoimmune processes [25–27]. On a global scale, the prevalence of type 1 diabetes is expected to rise from 8.4 million in 2021 to an estimated 13.5–17.4 million by 2040 [28], and the epidemic of obesity, which is growing in tandem, has major implications for type 1 diabetes. Type 1 diabetes incidence is rising in younger age groups, as well as in adults, with the shifting demographics to older populations [23]. Indeed, in 2022, 62% of new cases were diagnosed in people aged 20 years or older [23]. Obesity is becoming increasingly common in people with type 1 diabetes [13], particularly in high-income countries, where lifestyle contributes to weight gain. This overlap places a burden on healthcare and the healthcare economy, as type 1 diabetes management in the presence of obesity is more complex and costly (see Text box: Epidemiology of obesity and type 1 diabetes and scale of the problem). Type 1 and type 2 diabetes together account for a significant proportion of healthcare costs globally. Estimates suggest that, globally, the cost of diabetes care will exceed US$1 trillion by 2030, largely driven by the rising prevalence of obesity and its related complications [29, 30].


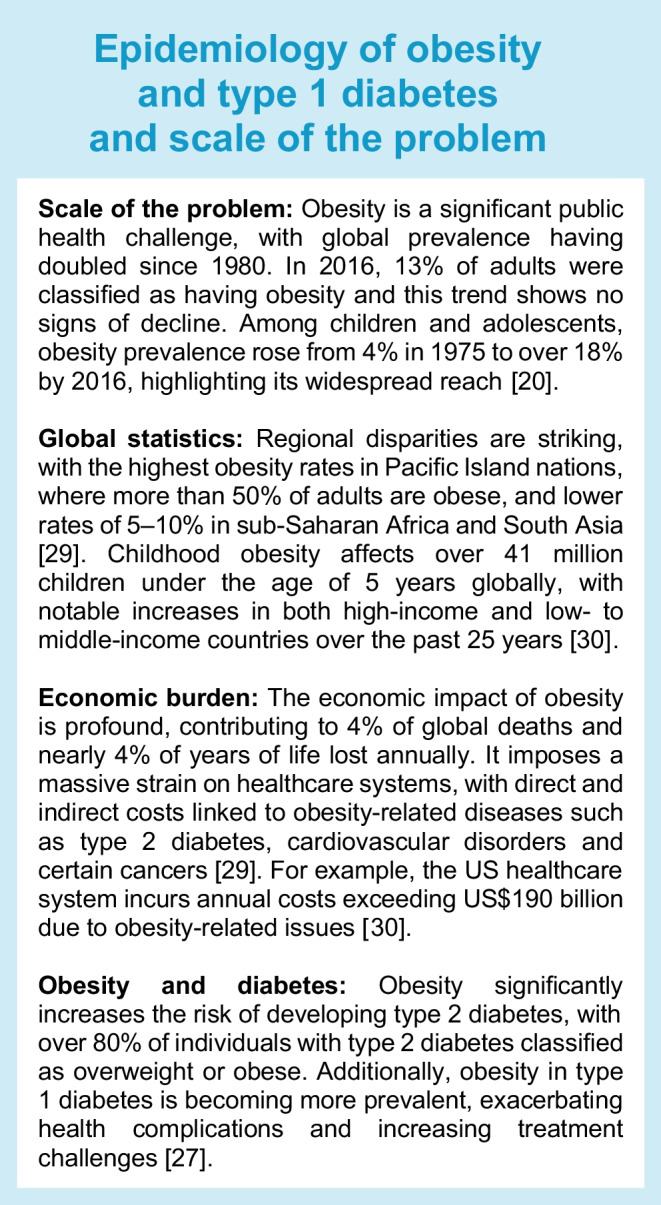


Obesity among individuals with type 1 diabetes has led to interest in ‘double’ or ‘dual’ diabetes, where features of type 1 and type 2 diabetes coexist [31]. Obesity predisposes to insulin resistance and to conditions such as hypertension, dyslipidaemia and steatotic liver disease. Obesity-related insulin resistance may lead to higher insulin requirements and further challenges around glucose management [26]. The resulting chronic hyperglycaemia can damage various organs, leading to microvascular complications such as retinopathy, nephropathy and neuropathy, as well as macrovascular complications such as CVD.

Insulin resistance plays a critical role in the cardiovascular risk profile of individuals with type 1 diabetes and obesity. Typically associated with type 2 diabetes, insulin resistance increases cardiovascular risk due to heightened inflammation, dyslipidaemia and hypertension. Individuals with type 1 diabetes and obesity have a higher prevalence of coronary artery disease, heart failure and other CVDs than those with type 1 diabetes and normal weight [32]. Management challenges thus also extend to managing cardiovascular risk, and other complications, in this population.

## Physiology and pathobiology of adipose tissue and its adaptation during obesity

Adipose tissue regulates energy balance through fat storage and use. There are two main types of adipose tissue: WAT and BAT. WAT primarily functions as an energy reservoir, storing triacylglycerol and playing endocrine roles by secreting hormones and cytokines, whereas BAT is involved in thermogenesis. Secreted hormones include leptin, which signals satiety and regulates energy balance; adiponectin, which enhances insulin sensitivity and counters inflammation; and resistin, which is associated with insulin resistance. Secreted cytokines include TNF and IL-6 [2, 33], with ATMs being the main source at tissue and systemic levels. Adipose tissue maintains metabolic stability by adapting to changes in energy availability, such as from increased caloric intake or during physical activity. In obesity, this balance is compromised and adipose tissue adapts by expanding. Adaptations include changes in cell size, number and composition, and limiting differentiation of beige adipocytes, which share phenotypic traits with BAT adipocytes (smaller lipid droplets, more mitochondria). Adipocyte hypertrophy leads to hypoxia because of insufficient vascularisation. Hypoxic and nutrient stress then trigger immune cell recruitment, including macrophages and monocytes, B and T lymphocytes, neutrophils, basophils, eosinophils, mast cells, dendritic and natural killer (NK) cells and innate lymphoid cells [2, 34] (Table 1).

**Table 1:** Immune cells, phenotypic markers and secreted molecules in obese visceral adipose tissue

Immune cells	Phenotypic markers	Secreted molecules
Macrophages		
M1-like ↑	F4/80, CD11b, CD11c	TNF ↑, IL-6↑, NOS2 ↑, IL-1β ↑
M2-like ↓	LYVE1, CD206, CD209, CD301	IL-10 ↓, IL-1Ra ↑, arginase ↑
Mast cells ↑	CD117, FCER1	Histamine ↑, PGE2 ↑, LTB4 ↑, TNF↑, IL-1β↑, IL-4 ↓, IL-6↑, IL-10 ↓, TGFβ ↑
Dendritic cells ↑	CD1c, CD11c, CD80, CD83, CD86	IL-12 ↑, IL-15 ↑
Neutrophils ↑	CD66b, CD11b, LY6G	Lysozyme ↑, NE ↑, MPO ↑, TNF ↑, IL-1β ↑, IL-8 ↑, MIP-1α ↑
Eosinophils ↑	CD45, SIGLEC8	IL-4 ↓, IL-10 ↓, IL-13 ↓, TGFβ ↑
Basophils ↑	CD9	IL-5 ↓
T lymphocytes		
Th1 ↑	CD4	IFN-γ ↑
Th2 ↓	CD4	IL-4 ↓, IL-5 ↓, IL-13 ↓
Th17 ↑	CD4	IL-17 ↑, IL-21 ↑, IL-22 ↑
Treg↓	CD4, CD25, FOXP3	IL-10 ↓, TGFβ ↑
B lymphocytes ↑	CD19, CD45R	IgG2c
NK cells ↑	CD3, NK1.1	TNF ↑, IFN-γ ↑, IL-4 ↓, IL-13 ↓
Innate lymphoid cells ↓	CD25	IL-5 ↓, IL-13 ↓

Arrows indicate changes in cell abundance or molecule expression or secretion in obesity compared with healthy conditions (↑ increased; ↓ decreased)

F4/80 (ADGRE1), adhesion G protein-coupled receptor E1; FCER1, Fc epsilon receptor Ia; FOXP3, forkhead box P3; IL-1Ra, IL-1 receptor antagonist; LTB4, leukotriene B4; LY6G, lymphocyte antigen 6 family member G; LYVE1, lymphatic vessel endothelial hyaluronan receptor 1; MIP-1α, C-C motif chemokine ligand 3; MPO, myeloperoxidase; NE, neutrophil elastase; NK1.1, natural killer cell lectin-like receptor subfamily B member 1 C; NOS2, nitric oxide synthase 2; PGE2, prostaglandin E2; SIGLEC8, sialic acid binding Ig like lectin 8; Th, T helper cell

ATMs are the most abundant immune cells in WAT. ATMs were historically categorised as M1 (classically activated, inflammatory) and M2 (alternatively activated, anti-inflammatory). This classification has expanded thanks to techniques allowing greater resolution of intermediate phenotypes and subpopulations (discussed below). M1-/M2-like states now represent polarisation extremes, with intermediate phenotypes on a sliding scale, which is useful when discussing ATMs en masse. At the M1-like extreme, ATMs express the marker CD11c and transcription factors IFN regulatory factor 3 (IRF3) and IFN regulatory factor 5 (IRF5), and produce cytokines/chemokines such as TNF, IL-6, IL-1β and CCL2 [33, 35]. M2-like ATMs express CD206 and release anti-inflammatory cytokines such as IL-10, TGF-β and IL-1 receptor antagonist. In lean states, ATMs constitute 10–15% of SVF and are M2-like while quiescent. In obesity, the number of ATMs increases and can constitute up to 40% of all WAT cells [36]. Most of these ATMs are proinflammatory. As obesity advances, M1-like macrophages accumulate, clustering around dead or dying adipocytes [37]. Of note, the number of adipose M2-like macrophages does not decrease in obesity [38]. However, obesity-associated factors, including NEFAs, lipid intermediates, lipopolysaccharides and hypoxia, shift the M1/M2 ratio to favour a proinflammatory state.

### ATM diversity and reclassification

Single-cell technologies have allowed greater resolution of ATM subtypes, expanding the M1-/M2-like paradigm. Alternatively activated macrophages are subclassified into perivascular or non-perivascular macrophages (PVMs, non-PVMs) [12]. PVMs express the markers CD206 and CD163 and the genes *Retnla* and *Lyve1* [12]. PVMs line vasculature to interact with endothelial cells, regulating vessel permeability, and their proportions decrease with obesity. PVMs also prevent pathogens from entering adipose tissue and can present antigens [12]. They can indirectly promote inflammation by expressing the chemokines CCL2, CCL3, CCL4 and CCL3L1, which attract monocytes [5]. Among non-PVMs are lipid-associated macrophages (LAMs), the adipose tissue foam cells with abundant intracellular lipids. LAMs are rare in lean WAT and the population expands with obesity; LAMs express CD9, triggering receptor expressed on myeloid cells 2 (TREM2) and CD63, and genes for lipid processing (e.g. *Lpl*, *Cd36*) [39]. This reclassification of ATMs has taken a functional perspective in single-cell studies. A meta-analysis of such studies reported four M2-like populations, a mixed M1-/M2-like population, a metabolically activated population, a redox-regulatory metabolic population and LAMs. Of these, LAMs and metabolically activated macrophages were associated with metabolic dysfunction in humans [40]. These macrophage subtypes have been reviewed elsewhere [41, 42].

### Other immune cells

**Fig. 1:**
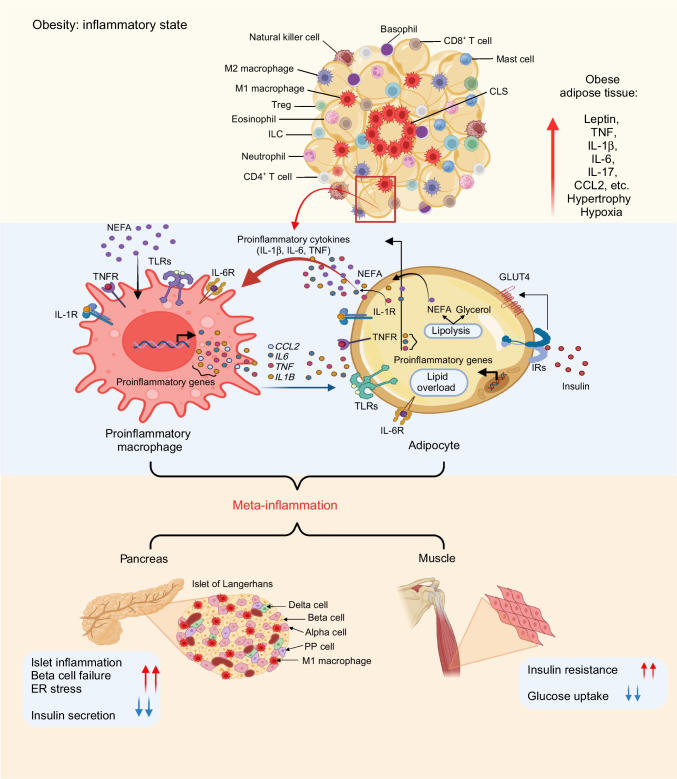
Obesity-driven adipocyte–immune cell miscommunication triggers inflammation, causing impaired insulin sensitivity and beta cell dysfunction. Obesity causes a shift in the populations of immune cells in adipose tissue, switching the usual quiescent state to a proinflammatory state with a predominance of ‘M1-like’ proinflammatory macrophages. The close interplay between adipocytes and immune cells, specifically proinflammatory macrophages, induces secretion of proinflammatory cytokines/chemokines and adipokines in a paracrine and/or endocrine fashion, resulting in a systemic proinflammatory state called meta-inflammation. This meta-inflammation interferes with insulin signalling, leading to insulin resistance in insulin target tissues, for example muscle. Within pancreatic islets, it promotes the infiltration of activated immune cells, resulting in islet inflammation, beta cell dysfunction, endoplasmic reticulum (ER) stress and impaired insulin secretion. CLS, crown-like structures; GLUT4, solute carrier family 2 (facilitated glucose transporter) member 4; IL-1R, IL-1 receptor type 1; IL-6R, IL-6 receptor; IR, insulin receptor; PP, pancreatic polypeptide; TLR, Toll-like receptor; TNFR, TNF receptor. Created in BioRender. Bahman, F. (2025) https://BioRender.com/nnc91vq. This figure is available as part of a downloadable slideset

Other leukocytes also shape the adipose tissue inflammatory environment. Neutrophils, mast cells, B cells and various T cell subsets (e.g. CD8^+^ T cells, CD4^+^ T helper 1 [Th1] cells) increase in abundance in obese WAT [34]. Increasing proportions of proinflammatory immune cells and their interactions with adipocytes result in ‘meta-inflammation’, which drives insulin resistance (Fig. 1). Several mechanisms contribute, including inhibitory phosphorylation of insulin receptor substrate (IRS) proteins, enhancing ceramide synthesis and adipocyte lipolysis, and inhibiting peroxisome proliferator-activated receptor gamma (PPAR-γ) expression. Notably, in obesity there is a decrease in the number of immune cells that control inflammation and restore insulin sensitivity, for example eosinophils, innate lymphoid cells (ILC2) and regulatory T cells (Tregs) [34]. The unique characteristics of visceral WAT Tregs are attributed to expression of PPAR-γ, which enhances insulin sensitivity [43]. Interestingly, M2-like ATMs also express PPAR-γ and promote insulin sensitivity [44]. Such mechanisms impact insulin signalling and insulin sensitivity [45].

### Cytokines, insulin production and insulin resistance

Insulin resistance disrupts glucose uptake and glycogen synthesis. To control blood glucose levels in insulin resistance, beta cells increase insulin secretion. More severe insulin resistance exacerbates beta cell stress, leading to beta cell exhaustion, hyperglycaemia and type 2 diabetes onset. The glucolipotoxic microenvironment exerts detrimental effects on beta cells through the action of proinflammatory factors (e.g. IL-6, IL-1β) released by beta cells and immune cells, particularly macrophages [46–48]. Additional studies have revealed that endotoxaemia and islet amyloid deposits also induce proinflammatory activation in islet macrophages, aggravating the cytokine-rich setting [49, 50]. Islet inflammation exacerbates beta cell dysfunction, triggering apoptosis and impairing glucose management.

IL-1β and IL-6 are of interest for their potency and physiological, sometimes reparative, actions. Chronic activation of the IL-1β system is deleterious in pancreatic islets and insulin target tissues, disrupting insulin production (beta cell death) and causing insulin resistance [51]. IL-1β also plays physiological roles in glucose metabolism [52]. Peritoneal macrophages postprandially secrete IL-1β in a glucose-dependent manner, contributing to postprandial insulin secretion [10]. Abolishing endogenous IL-1β signalling in mice diminishes postprandial plasma insulin levels. Part of this mechanism suggests that increased glucose uptake by macrophages reinforces their proinflammatory profile, a state limited by efficient blood glucose normalisation [52]. For IL-6, divergence of beneficial and deleterious roles may be explained by its source (IL-6 also exists as an exercise-induced myokine) and/or exposure dose and duration [53, 54]. In murine models of tissue-specific gene knockout, myeloid *Il6* deficiency worsens inflammation and metabolic outcomes in obesity; adipose *Il6* deficiency dampens inflammation and improves metabolic outcomes; and muscle *Il6* deficiency has no influence on metabolic outcomes [10]. In humans with type 2 diabetes, circulating insulin is decreased when recombinant IL-6 is acutely administered. Clamp studies in healthy participants suggest that this is due to insulin sensitisation in peripheral tissues [53]. While these cytokines have been extensively studied in type 2 diabetes, their contributions to type 1 diabetes are understudied. Circulating IL-6 and IL-1β levels are elevated in individuals with type 1 diabetes compared with those without diabetes, with higher IL-1β levels in early-stages and with worse management [55, 56].

### Chemokines

Chemokines, which recruit leukocytes, also shape the inflammatory microenvironment, contributing to WAT inflammation and beta cell destruction. Among these, CCL2 and CXCL10 are most relevant. CCL2 recruits monocytes and macrophages into inflamed tissues, including islets. Beta cells upregulate CCL2 expression in response to proinflammatory cytokines, facilitating immune cell recruitment and amplifying insulitis [57]. Elevated CCL2 has been detected in pancreatic tissues during type 1 diabetes progression, highlighting its role in islet inflammation. Similarly, CXCL10 is expressed in islets during early disease stages and is instrumental in recruiting autoreactive T cells via the CXCR3 receptor [58]. Blocking CXCL10 reduces immune infiltrate, delaying type 1 diabetes onset in experimental models [59].

Obesity-associated chemokines strengthen the link between metabolic and immune dysfunction in type 1 diabetes. WAT secretes CCL2 and CXCL10, contributing to systemic inflammation. Adipose chemokine secretion promotes immune cell trafficking to pancreatic islets, sustaining an inflammatory milieu that fragilises islets, potentially exacerbating autoimmunity [60, 61]. Given the increasing prevalence of obesity in type 1 diabetes, deciphering how chemokines bridge metabolic stress and immune activation is essential.

### Mechanisms of influence of obesity on the pathogenesis of type 1 diabetes

Obesity can disrupt self-tolerance through chronic low-grade inflammation, decreasing Tregs and increasing Th17/Th1 populations and favouring the development of autoimmune disorders [62]. Childhood obesity is associated with higher type 1 diabetes susceptibility [47]. Overweight children with new-onset type 1 diabetes have adipokine and cytokine profiles consistent with heightened inflammation, potentially contributing to type 1 diabetes onset [63]. Insulin resistance from obesity-driven meta-inflammation will also complicate glucose management.

**Fig. 2:**
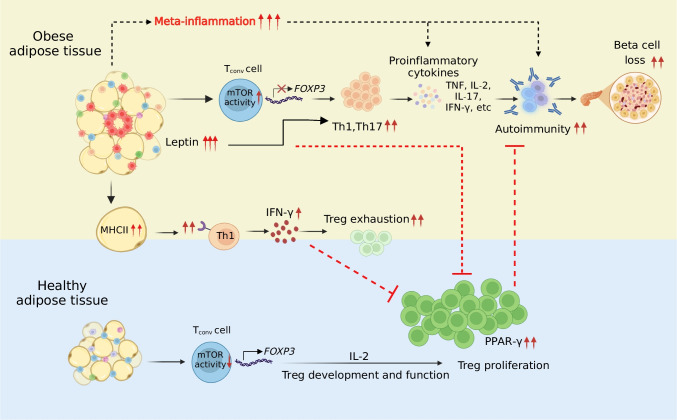
Mechanistic links between obesity and autoimmunity. In obesity, adipose tissue inflammation (meta-inflammation) disrupts immune regulation through several mechanisms: persistent mammalian target of rapamycin (mTOR) activation in T cells reduces regulatory T cell (Treg) differentiation; elevated leptin levels impair Treg function and enhance pro-inflammatory Th1 and Th17 cell activity; increased expression of MHC class II in adipocytes promotes CD4+ T cell activation and IFN-γ secretion, further exhausting Tregs; and diminished PPAR-γ activity compromises Treg proliferation. Collectively, these alterations promote autoimmune responses leading to pancreatic beta cell loss characteristic of type 1 diabetes. Healthy adipose tissue supports *FOXP3* expression, promoting the development and maintenance of anti-inflammatory Tregs. Created in BioRender. Bahman, F. (2025) https://BioRender.com/imbg578. This figure is available as part of a downloadable slideset

A prospective cross-sectional cohort study of youth with type 1 diabetes demonstrated that central obesity is associated with earlier onset [64]. Higher BMI at birth has also been associated with an increased risk of type 1 diabetes in children. Epidemiological studies show that type 1 diabetes incidence rises almost linearly with birthweight. A reduction in type 1 diabetes should be considered a potential additional benefit of obesity prevention [18]. From a mechanistic perspective, five key lines of evidence (Fig. 2) suggest that WAT may play a role in driving autoimmune pathogenesis in type 1 diabetes.

First, in obesity, chronic exposure to leptin and nutrient surpluses leads to persistent activation of the mammalian target of rapamycin (mTOR) signalling pathway in T cells, disrupting normal T cell receptor (TCR)-mediated signalling. This overactivation creates an environment resembling supra-physiological TCR stimulation, which does not favour *FOXP3* gene transcription in conventional CD4^+^CD25^−^ T cells (T_conv_: CD4^+^CD25^−^FOXP3^−^). The transcription factor forkhead box P3 (FOXP3) is crucial for the development and maintenance of anti-inflammatory CD4^+^CD25^+^FOXP3^+^ Tregs. Consequently, obesity negatively affects both conversion of T_conv_ cells into Tregs in the periphery and proliferation of thymically-derived Tregs, impairing their immune regulatory influence [62].

Second, leptin inhibits the proliferation and function of Tregs and sustains the proliferation, activation and proinflammatory functions of Th1/Th17 cells [65, 66]. This transformation heightens risk of disrupted immunological self-tolerance, contributing to various autoimmune diseases and chronic inflammation.

Third, adipocytes in obese WAT increase expression of MHC class II genes, allowing them to present antigens to activate CD4^+^ T cells [67]. This heightened antigen-presenting capacity leads Th1 cells to secrete high levels of IFN-γ, a proinflammatory cytokine. Elevated IFN-γ levels disrupt immune homeostasis by inhibiting the differentiation of Tregs and inducing their loss, likely through mechanisms of cellular exhaustion [67].

Fourth, Th17-driven inflammatory responses are caused by diminished activity of PPAR-γ. Furthermore, PPAR-γ expression in WAT is essential for the differentiation and functionality of resident Tregs, suggesting a reciprocal connection between WAT dynamics and immune tolerance mechanisms mediated by Tregs [43].

Fifth, meta-inflammation within obese WAT heightens risk of peripheral tissue damage and autoimmunity. Metabolic workload, driven by nutrient excess, adipocyte-secreted growth factors and proinflammatory cytokines, amplifies autoimmune pathogenesis, especially in the context of a Western diet high in energy-dense, nutrient-poor foods.

## Immune dysfunction in type 1 diabetes and the impact of obesity

### Autoimmunity

Immune dysfunction in type 1 diabetes refers to beta cell-directed autoimmunity, classically seen as an offensive attack from CD8^+^ T cells that detect autoantibodies from insulin-producing beta cells. This linear explanation has evolved to include other hypotheses, including that beta cells themselves experience and signal their own biosynthetic stress [68]. Stress signals emanating from beta cells result in immune-mediated targeting of the source of insulin, making beta cells sources of dysfunction alongside the immune system. Nevertheless, islet autoimmunity remains an essential component of pathogenesis; only the driving factors and subsequent immune adaptations are modified in the above hypothesis. The relevance of these developing points of view, which extend previously described mechanisms, is that they leave room to develop hypotheses on mechanisms that interact with obesity.

There is overwhelming evidence that type 1 diabetes is a T cell-mediated autoimmune condition [69]. In 1975, Cudworth and Woodrow first described an association of HLA antigens with juvenile-onset and/or insulin-dependent, but not insulin-independent, forms of diabetes [70]. They reported that the HLA chromosomal region that controls immune responses may be involved in promoting disease susceptibility [70]. The contribution of HLA genes to the genetic risk of type 1 diabetes has subsequently been confirmed multiple times and solidified in the era of genome-wide association to explain up to 50% of the risk [71]. Functionally, excessive genetic risk is conferred by dendritic cell presentation of islet epitopes to shape adaptive immune responses and initiate autoantibody production [72]. Seroconversion marks the onset of autoimmunity and is the current best predictor for loss of tolerance and clinical manifestation of type 1 diabetes.

Immune cells infiltrate pancreatic islets, creating an inflammatory microenvironment, leading to insulitis and selective destruction of insulin-producing beta cells. Given the above description, autoreactive T cells unequivocally form a part of type 1 diabetes pathogenesis. However, multiple findings suggest that our understanding remains incomplete. These include that autoreactive T cells, while present at a higher density in the pancreases of individuals with type 1 diabetes, do not differ in circulating frequencies between those with type 1 diabetes and those without [73]. Another observation is that Tregs, renowned for maintaining tolerance by suppressing autoreactivity, lose their suppressive capacity in type 1 diabetes without any effect on their frequency [74]. In addition, the transferability of type 1 diabetes by bone marrow transplantation from an individual with insulin-dependent diabetes to an HLA-identical recipient, who was autoantibody negative at the time of donation, indicates that non-beta cell-related and non-HLA-related factors may dictate susceptibility [75].

### Autoimmunity affected by obesity

Metabolic overload in obesity is sensed by the body’s component cells, including beta cells and immune cells. Immunometabolic mechanisms that sense and respond to metabolic status at the systemic, microenvironmental or cellular level are now considered central to controlling immune cell function.

The lines of evidence tying obesity to autoimmunity are numerous in type 1 diabetes and other autoimmune conditions [62]. In type 1 diabetes, increased risk due to higher body weight is present from birth, with higher BMI at birth associated with higher susceptibility to type 1 diabetes [76]. Other studies have found similar associations for islet autoantibody seroconversion and beta cell function in neonates and infants, as well as parental type 1 diabetes [77, 78]. No differences were found in early-life beta cell function between children who seroconverted and those who did not [77]. Higher birthweight is associated with seroconversion risk, although it is also associated with maternal type 1 diabetes. Children born to mothers with type 1 diabetes have a lower risk of seroconversion than children born to fathers with type 1 diabetes, with children born to fathers with type 1 diabetes not having a higher birthweight [77, 78]. These studies confirm that birthweight contributes to metabolic stress and modifies risk of type 1 diabetes. They also confirm that beta cell workload per se is not a risk factor for islet-directed autoimmunity, and that other factors related to parental type 1 diabetes modify risk.

Other studies have addressed BMI and pancreatic workload in older participants. In one study, the pancreases of individuals at risk of type 1 diabetes were the same size and weight as those of individuals diagnosed with type 1 diabetes. Both at-risk and diagnosed groups had smaller pancreases than individuals without risk or diagnosis of type 1 diabetes, after adjusting for age and BMI [79]. Another study focused on excess BMI in the TrialNet Pathway to Prevention cohort (autoantibody-positive relatives of individuals with type 1 diabetes who do not have diabetes themselves) [80]. Here, longitudinally accumulated excess BMI increased the risk of type 1 diabetes in older male (>35 years) and younger female (<35 years) participants [80]. This indicates that overweight and obesity contribute to the risk of developing type 1 diabetes in those already at risk, and that risk is modified by sex.

A Mendelian randomisation from the UK Biobank indicated that increased risk of type 1 diabetes is not a special case in obesity; rather, there is also an increased risk of other autoimmune conditions [15]. In this study, a 1 SD increase in genetically predicted BMI increased the odds of type 1 diabetes by 1.55-fold; causal links were also made with asthma, hypothyroidism, psoriasis and rheumatoid arthritis [15].

The notion of metabolic stress on islets, brought on by functional fatigue due to early-life events or by obesity later in life, puts into perspective the role of autoimmunity. Indeed, the presence of autoreactive T cells is not a prerequisite for the development of type 1 diabetes, as the population without type 1 diabetes also harbours autoreactive T cells, meaning these cells form part of a physiological immune repertoire [73]. In addition, only 10% of individuals with islet autoantibodies ever develop type 1 diabetes [81–83]. Globally, these observations indicate that islet-directed immune responses may be the consequence of other mechanisms impacted by obesity.

Supporting a genetically and environmentally reactive role for autoimmunity, Warncke et al found that, in 1050 infants and children (aged 4 months to 3.6 years) with a high genetic risk of type 1 diabetes, glycaemic trajectories were determined by sex, BMI, glucose-related genetic risk score and the type 1 diabetes-susceptible *INS* genotype. Postprandial blood glucose increased and remained high about 2 months prior to autoantibody positivity in children who seroconverted. This study provides an alternative paradigm for type 1 diabetes pathogenesis, that is, heterogeneity in glycaemic control precedes islet autoimmunity, and this may be influenced by genetic and environmental factors.

## Clinical and translational implications

### Diabetes risk and staging

Obesity-related factors that increase metabolic burden, impacting systemic inflammation and the autoimmune response, are as relevant to the presymptomatic stages of type 1 diabetes as they are in clinically established type 1 diabetes. Type 1 diabetes staging proposed by major organisations refers to three stages: (1) normoglycaemic with two or more autoantibodies; (2) dysglycaemic with two or more autoantibodies; (3) dysglycaemic with two or more autoantibodies and symptoms of diabetes, which may include polydipsia, polyuria, fatigue and diabetic ketoacidosis [84]. The scientific statement detailing these stages also includes prestaging, which considers genetic risk linked to the HLA region; this is particularly relevant in research settings aiming to establish predictors or confounding mechanisms, as in the study by Warncke et al [85]. More recent studies by Haynes et al. and Desouter et al. employed continuous glucose monitoring (CGM) metrics to detect early dysglycaemia in at-risk and seroconverted children [86, 87]. These studies highlight how uptake of CGM devices adds value to research and clinical practice in detecting, monitoring and predicting progress through type 1 diabetes staging. The study by Desouter et al, in particular, shows that pairing CGM metrics with HbA_1c_ is nearly as effective as repeated glucose tolerance testing in predicting progression to stage 3 type 1 diabetes, providing a more convenient tool for longitudinal monitoring [87].

### Management and lifestyle

**Fig. 3:**
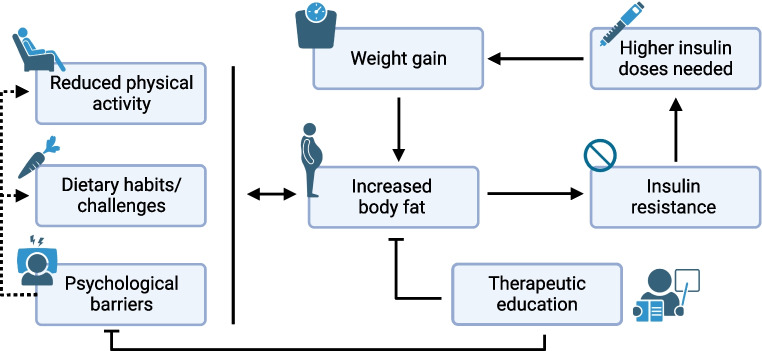
Obesity and management challenges in type 1 diabetes. The cyclical relationship between increased body fat, insulin resistance, higher insulin doses and weight gain highlights how excess adiposity contributes to a cascade of physiological and behavioural factors that complicate type 1 diabetes management. Created in BioRender. Alzaid, F. (2025) https://BioRender.com/3hiviw3. This figure is available as part of a downloadable slideset

Coexistence of obesity and type 1 diabetes presents unique challenges for management (Fig. 3). Obesity contributes to insulin resistance, which necessitates higher insulin doses to maintain glucose levels [88]. This increased insulin requirement can create a cycle whereby insulin therapy promotes further weight gain, exacerbating obesity and further complicating glucose management [89, 90]. Therapeutic education becomes more complex as individuals must navigate dietary modifications that address both weight and glucose management.

Behavioural modifications are also more challenging in individuals with obesity and type 1 diabetes. Increased adiposity can reduce mobility and exercise tolerance, making it harder to incorporate daily physical activity. However, there have been successful examples of lifestyle modification that improve treatment targets for both type 1 diabetes and obesity [91, 92]. Additionally, obesity-related psychological factors, such as reduced self-esteem, depression and disordered eating, may hinder adherence to treatment plans or compound the psychological burden of living with diabetes, such as diabetes distress, which is more common in type 1 diabetes and is associated with poorer outcomes [93–95].

Precision medicine enables personalised treatment for individuals with obesity and type 1 diabetes. Such management considers genetic, metabolic and lifestyle factors to optimise therapy [96]. For instance, insulin regimens can be adjusted based on individual insulin sensitivity, reducing hypoglycaemia risk and minimising weight gain. Health technologies such as CGMs and automated insulin delivery (AID) systems provide real-time data and feedback, allowing for precise insulin dosing and better glucose management [97, 98]. Advanced algorithms in closed-loop systems can reduce the risk of hypoglycaemia or hyperglycaemia and, over time, will provide valuable information on the management of obesity and type 1 diabetes [99, 100]. Mobile health applications provide platforms for tracking diet, physical activity and blood glucose, facilitating engagement and self-management [101].

Nutritional interventions should promote weight loss without compromising glucose management. Diets emphasising low glycaemic index foods can help manage postprandial glucose spikes while supporting weight management [102]. Behavioural therapies, such as motivational interviewing and cognitive behavioural techniques, can also enhance adherence to dietary and exercise recommendations [103].

### Repositioning therapies

Therapies for type 2 diabetes have been explored for potential benefits in individuals with type 1 diabetes and obesity. Glucagon-like peptide-1 receptor agonists and sodium–glucose cotransporter 2 inhibitors have shown promise in promoting weight loss and improving glucose management [104, 105]. However, their use in type 1 diabetes requires careful monitoring because of the risk of ketoacidosis [105]. Recent and ongoing trials aim to assess the efficacy of these agents in type 1 diabetes, with a focus on those with obesity (NCT03878459, [106]).

Obesity is associated with chronic low-grade inflammation, which can exacerbate insulin resistance and may influence autoimmune processes. Therapeutically targeting immune–adipose interactions provides a novel approach to improving management [106, 107]. Anti-inflammatory agents that modulate cytokine activity could potentially reduce insulin resistance and preserve beta cell function.

Research is also exploring the role of adipokines in inflammation and autoimmunity. For example, increasing levels of adiponectin, which has anti-inflammatory effects, might improve insulin sensitivity [108]. Lifestyle interventions that reduce adiposity can also decrease proinflammatory cytokine levels, mitigating obesity-related inflammation [109].

### Complication risk

Obesity increases the risks of CVD, nephropathy and other complications in individuals with type 1 diabetes [110]. Improved risk stratification enables early identification of high-risk individuals and the implementation of targeted interventions. Biomarkers such as C-reactive protein, IL-6 and TNF can indicate the presence of systemic inflammation associated with obesity [111]. Moreover, use of cytometry-based immunophenotyping is proving to be a precise and useful tool, reflecting systemic inflammation and indicating cardiovascular risk in diabetes [112] Cardiovascular risk, in particular, is a special case in type 1 diabetes, as only a portion of the risk is accounted for by classical risk factors. It has been demonstrated that part of the type 1 diabetes-specific risk is due to cardiac-specific autoimmunity [113]. An independent specific risk prediction tool, the Steno T1 Risk Engine, has been developed to screen for cardiovascular risk in people with type 1 diabetes [114]. Immunophenotyping approaches, either stand-alone or combined with other predictors, have potential to improve risk prediction; however, they are yet to be fully exploited in this context.

## Emerging concepts and future directions

Translational models have advanced our understanding of type 1 diabetes and its interactions with obesity. The NOD mouse model is widely used to study autoimmune diabetes because of its spontaneous development of type 1 diabetes-like symptoms [115]. However, NOD mice are inherently lean, which limits their applicability in obesity research. To address this, researchers have developed hybrid models by crossing NOD mice with obese strains or inducing obesity through high-fat diets [116]. In streptozocin-induced diabetes models, pancreatic beta cell destruction mimics type 1 diabetes [117]. While streptozocin models can incorporate obesity, they lack autoimmunity, limiting their translational relevance. Table 2 provides a summary of the translatability of the above models and others commonly used in diabetes research.

**Table 2:** Translatability of type 1 diabetes, type 2 diabetes and obesity models

Model	Application	Strengths	Limitations
NOD mouse	T1D: autoimmune diabetes research	Spontaneous T1D development mimics human autoimmune mechanisms	Inherently lean; lacks relevance to obesity-associated diabetes [125]
Streptozocin-induced models	T1D: beta cell destruction, mimics diabetes onset	Reproducible beta cell loss and hyperglycaemia	Lacks autoimmune component; effects induced chemically rather than immunologically [126]
*db*/*db* mouse	T2D: obesity-associated insulin resistance and hyperglycaemia	Exhibits obesity, hyperinsulinaemia and insulin resistance	Does not exhibit autoimmune features of T1D; exclusively T2D-focused [127]
*ob*/*ob* mouse	T2D/obesity: studies leptin deficiency-induced obesity and related metabolic syndromes	Key model for leptin biology and energy regulation research	Leptin deficiency does not mimic common human obesity mechanisms [128]
Diet-induced obesity models	Obesity/T2D: mimics high-fat diet-induced obesity and metabolic dysfunction	Models dietary and environmental influences on obesity and T2D	Highly variable outcomes depending on diet composition, strain and duration of feeding [129]
High-fat diet in NOD mice	T1D/obesity: combines obesity and autoimmune diabetes	Provides insights into obesity’s role in autoimmune diabetes progression	Limited standardisation of protocols; variability in obesity induction among NOD strains [130]
Zucker diabetic fatty rat	T2D/obesity: studies the progression from obesity to T2D	Exhibits obesity, dyslipidaemia and glucose intolerance	Genetic mutations limit applicability to polygenic human obesity and T2D [131, 134]
Humanised mouse models	T1D/obesity: human immune system transplantation for personalised research	Allows study of human-specific immune and metabolic responses	Complex and costly; immune responses may not fully replicate human pathophysiology [133]
Pig and non-human primate models	T1D/T2D/obesity: large animal models for advanced translational research	Physiological similarities to humans; useful for surgical and pharmacological intervention studies	Ethical and logistical challenges; limited genetic tools and long gestation times [134]

T1D, type 1 diabetes; T2D, type 2 diabetes

Future research should focus on developing more representative translational models that encompass autoimmune and metabolic aspects of type 1 diabetes and of obesity. This includes creating humanised mouse models or using induced pluripotent stem cells to study disease mechanisms in a human context [118].

The era of big data and technological innovations is transforming type 1 diabetes research. Single-cell genomics allows for detailed characterisation of immune cells and pancreatic islet cells at the individual cell level [119, 120]. This technology has uncovered heterogeneity in immune cell populations and identified novel cell subsets involved in autoimmunity and inflammation.

Digital health technologies, including wearable devices and mobile applications, generate vast amounts of real-world data on glucose levels, physical activity and other metrics [121]. Machine learning algorithms can analyse this data to identify patterns and predict disease progression or treatment responses. Integrating digital data, multi-omics data—genomics, proteomics and metabolomics—with clinical information can lead to a more comprehensive understanding of obesity and type 1 diabetes.

There is also a need to harness technological advances for precision medicine. Large-scale collaborative initiatives should focus on integrating multi-omics data with clinical outcomes to uncover biomarkers for disease risk stratification and identify potential therapeutic targets. Investigating the role of the gut microbiome in immune regulation and metabolism may also reveal novel intervention options [122–124].

## Conclusion

The relationship between obesity and type 1 diabetes is complex, with intersecting metabolic and autoimmune processes that impact pathogenesis and management. Historically considered to affect lean individuals, type 1 diabetes is increasingly co-occurring with obesity, a trend mirroring the global rise in obesity rates. This convergence complicates management because of insulin resistance and introduces additional cardiovascular and metabolic risks.

WAT is an endocrine organ that influences immune function. The expansion and maladaptation of WAT in obesity leads to chronic low-grade inflammation, with infiltration of proinflammatory immune cells and secretion of proinflammatory cytokines. Meta-inflammation contributes to insulin resistance and may play a role in the initiation and progression of autoimmunity in type 1 diabetes. Mechanistic insights reveal that obesity-induced alterations in immunity, including impaired function of Tregs and enhanced activity of proinflammatory Th cells, could potentiate autoimmune responses against beta cells.

Clinically, obesity in individuals with type 1 diabetes necessitates individualised management. Insulin therapies may inadvertently promote weight gain, creating a vicious cycle that complicates metabolic control (Fig. 3). Advances in health technology, such as CGM and AID systems, provide promising tools to enhance management while mitigating weight-related challenges. Moreover, repurposing medications from type 2 diabetes presents potential therapeutic avenues, although their use requires careful monitoring because of associated risks.

Future directions emphasise the importance of developing translational models that reflect the dual metabolic and autoimmune dimensions of type 1 diabetes complicated by obesity. Technological advancements in single-cell genomics and digital health can deepen our understanding of disease mechanisms and support development of precision medicine. Integrating multi-omics data with clinical information could identify novel biomarkers for risk stratification and new therapeutic targets.

In summary, addressing obesity and type 1 diabetes is increasingly necessary to improve patient outcomes. A comprehensive approach combining mechanistic research, technological innovation and personalised management holds promise of mitigating the compound risks of both conditions. By recognising and targeting the challenges presented, healthcare providers can enhance management and ultimately improve quality of life and lower disease burden.

## Supplementary Information

Below is the link to the electronic supplementary material.

ESM Text box (PDF 141 KB)

Slideset of figures (PPTX 715 KB)

## Abbreviations

AID: Automated insulin delivery
ATM: Adipose tissue macrophage
BAT: Brown adipose tissue
CGM: Continuous glucose monitoring
LAM: Lipid-associated macrophage
NK: Natural killer
PPAR-γ: Peroxisome proliferator-activated receptor gamma
PVM: Perivascular macrophage
SVF: Stromal vascular fraction
Treg: Regulatory T cell
WAT: White adipose tissue
