# Supplementary material for: Immune cell–adipose tissue crosstalk in metabolic diseases with a focus on type 1 diabetes
Source: Diabetologia. 2025 Jun 4;68(8):1616–31. doi: 10.1007/s00125-025-06437-z (PMC12246022; doi:10.1007/s00125-025-06437-z)
Supplement: Supplementary file 1 — ESM Text box (PDF 141 KB) [file 125_2025_6437_MOESM1_ESM.pdf]

Key facts on obesity

BMI grading and classes

- Normal weight: 18.5–24.9 kg/m²
- Overweight: 25.0–29.9 kg/m²
- Obesity:
  - class I: 30.0–34.9 kg/m²
  - class II: 35.0–39.9 kg/m²
  - class III (severe): ≥40.0 kg/m²

Physiopathology

- Excess lipids: adipocyte hypertrophy leads to lipid storage dysfunction and ectopic fat deposition.
- Ectopic lipid accumulation: lipid deposition in liver, muscle and pancreas contributes to insulin resistance.
- Chronic inflammation: adipose tissue secretes proinflammatory cytokines (e.g. TNF, IL-6) that drive systemic inflammation.
- Insulin resistance: chronic low-grade inflammation disrupts insulin signalling pathways, promoting insulin resistance and glucose intolerance.

Common complications

- Metabolic: type 2 diabetes, dyslipidaemia and metabolic dysfunction-associated steatotic liver disease (MAFLD).
- Cardiovascular: hypertension, atherosclerosis and coronary artery disease.
- Mechanical: obstructive sleep apnoea and osteoarthritis.
- Psychosocial: depression and reduced quality of life.

Common treatments

- Lifestyle interventions: caloric restriction, physical activity and behavioural therapy.
- Pharmacological: glucagon-like peptide-1 receptor agonists and sodium–glucose cotransporter 2 inhibitors.
- Surgical: bariatric surgery for severe obesity.
- Emerging approaches: anti-inflammatory therapies targeting cytokine pathways.
