# Supplementary material for: Immune cell–adipose tissue crosstalk in metabolic diseases with a focus on type 1 diabetes
Source: Diabetologia. 2025 Jun 4;68(8):1616–31. doi: 10.1007/s00125-025-06437-z (PMC12246022; doi:10.1007/s00125-025-06437-z)
Supplement: Supplementary file 2 — Slideset of figures (PPTX 715 KB) [file 125_2025_6437_MOESM2_ESM.pptx]

## Slide 1
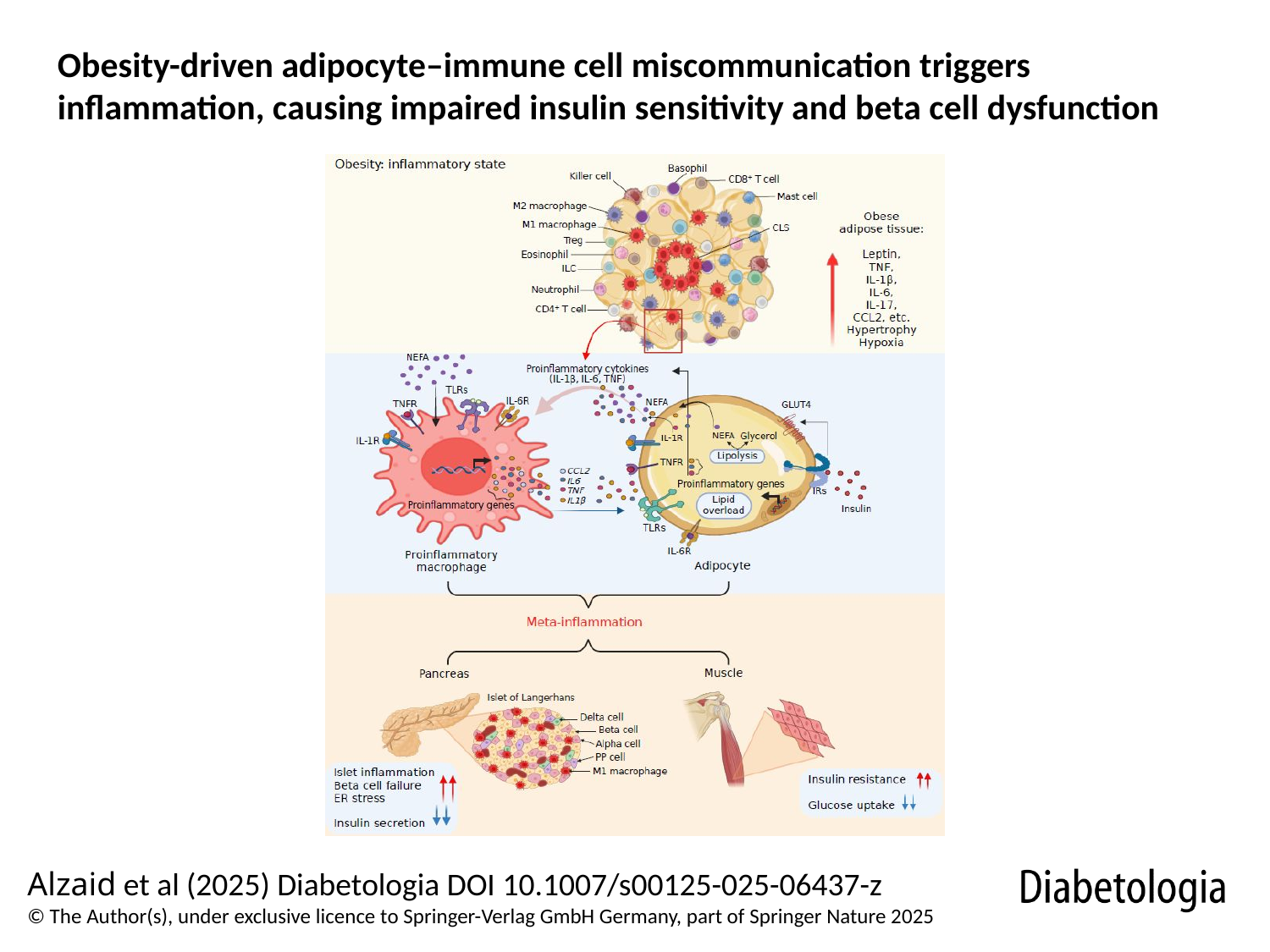

Obesity-driven adipocyte–immune cell miscommunication triggers inflammation, causing impaired insulin sensitivity and beta cell dysfunction
Alzaid et al (2025) Diabetologia DOI 10.1007/s00125-025-06437-z
© The Author(s), under exclusive licence to Springer-Verlag GmbH Germany, part of Springer Nature 2025

## Slide 2
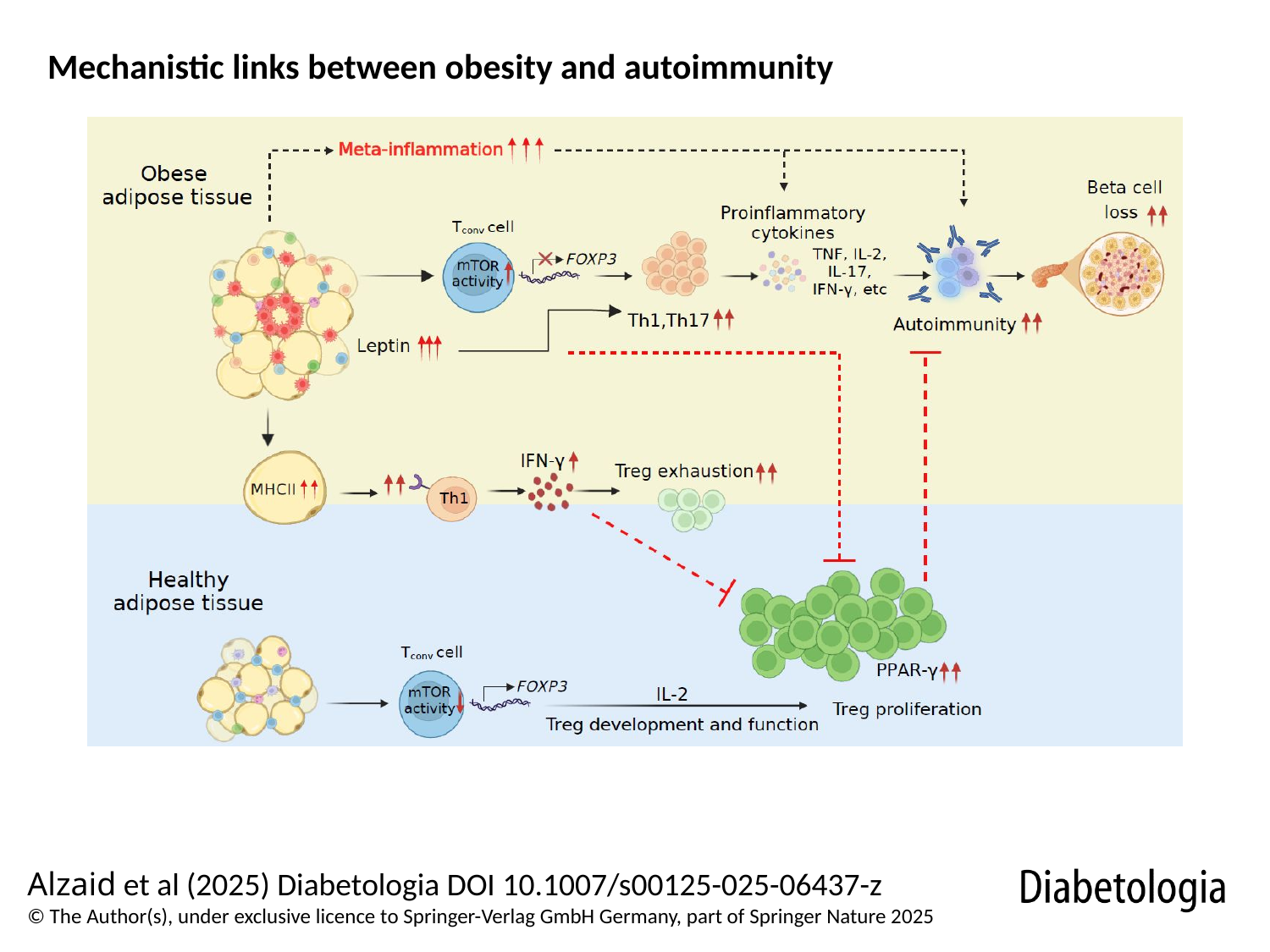

Mechanistic links between obesity and autoimmunity
Alzaid et al (2025) Diabetologia DOI 10.1007/s00125-025-06437-z
© The Author(s), under exclusive licence to Springer-Verlag GmbH Germany, part of Springer Nature 2025

## Slide 3
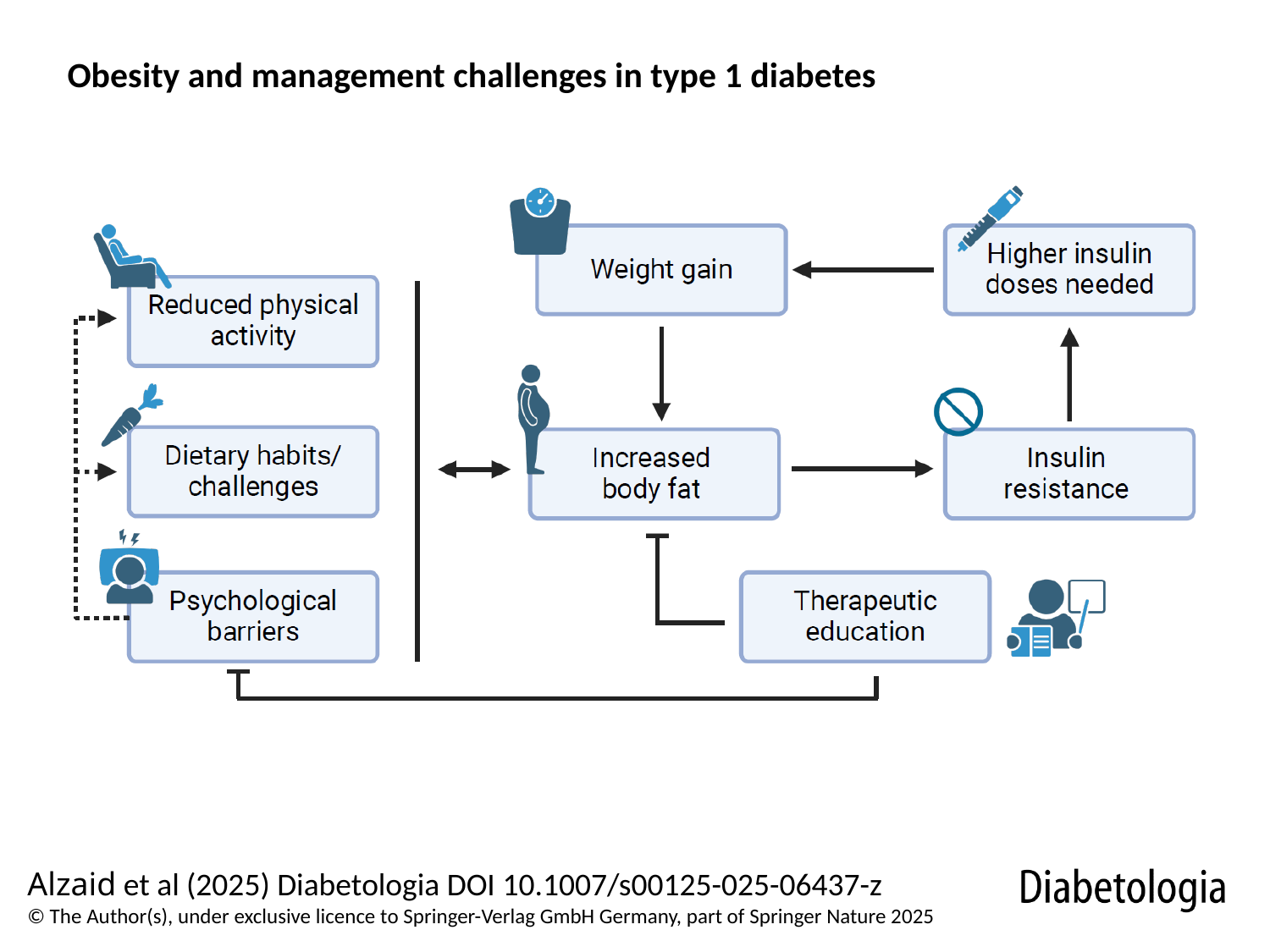

Obesity and management challenges in type 1 diabetes
Alzaid et al (2025) Diabetologia DOI 10.1007/s00125-025-06437-z
© The Author(s), under exclusive licence to Springer-Verlag GmbH Germany, part of Springer Nature 2025
